# Microstructural Hippocampal Alterations in Alzheimer's Disease: A Systematic Review and Meta‐Analysis of Diffusion Kurtosis Imaging

**DOI:** 10.1002/brb3.70919

**Published:** 2025-09-21

**Authors:** Amir Mahmoud Ahmadzadeh, Sadegh Ghaderi, Sana Mohammadi, Nahid Jashirenezhad, Farzad Fatehi

**Affiliations:** ^1^ Department of Radiology, School of Medicine Mashhad University of Medical Sciences Mashhad Iran; ^2^ Neuromuscular Research Center, Department of Neurology Shariati Hospital Tehran University of Medical Sciences Tehran Iran; ^3^ The Persian Gulf Tropical Medicine Research Center, The Persian Gulf Biomedical Sciences Research Institute Bushehr University of Medical Sciences Bushehr Iran

**Keywords:** Alzheimer's disease | biomarker | diffusion kurtosis imaging | hippocampus | mean kurtosis

## Abstract

**Background:**

The hippocampus is highly vulnerable in Alzheimer's disease (AD), with early microstructural changes potentially detectable via diffusion kurtosis imaging (DKI). Previous studies report promising DKI findings in AD, necessitating systematic evaluation. To compare hippocampal DKI metrics, particularly mean kurtosis (MK), between AD patients and healthy controls (HCs) and explore factors influencing these differences.

**Methods:**

Following PRISMA guidelines, PubMed, Scopus, Web of Science, and Embase were searched until November 2024. Two reviewers independently extracted hippocampal MK values. Risk of bias was evaluated using the Newcastle–Ottawa Scale. Meta‐analysis employed random‐effects models (STATA v17). Subgroup analyses (sex, age, magnetic resonance imaging [MRI] parameters) and sensitivity and trim‐and‐fill assessments were conducted. Standardized mean difference (SMD), *I* ^2^ for heterogeneity, and Egger's/Begg's tests for publication bias (significance: *p* < 0.05).

**Results:**

Ten studies (215 AD patients, 217 HCs; mean age: 65–75 years) using 3.0 T MRI were included. Eight articles were included in the meta‐analysis to compare MK between groups. AD patients exhibited significantly reduced bilateral hippocampal MK compared to HCs (left: SMD = −1.32, 95% confidence interval [95% CI] [−1.97 to −0.66]; right: SMD = −1.22 [−1.88 to −0.56]; both *p *< 0.001), indicating compromised microstructural complexity. Subgroup analyses revealed more pronounced MK reductions in studies with higher male ratios (>42%; left: SMD = −1.87; right: SMD = −1.91; *p* < 0.05). Age, echo time, repetition time, and diffusion directions did not significantly influence effect sizes. Sensitivity analyses confirmed robustness, and publication bias was detected, but trim‐and‐fill analyses revealed no missing studies.

**Conclusion:**

Reduced hippocampal MK in AD reflects microstructural degeneration, with sex‐related differences in effect magnitude.

## Graphical abstract

This meta‐analysis of 10 studies reveals that individuals with Alzheimer's disease have significantly reduced hippocampal mean kurtosis, indicating compromised microstructural complexity. These changes were more pronounced in studies with a higher proportion of male participants, suggesting sex‐specific differences in hippocampal vulnerability.

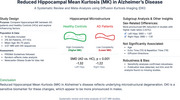

## 1 Introduction

**FIGURE 1:**
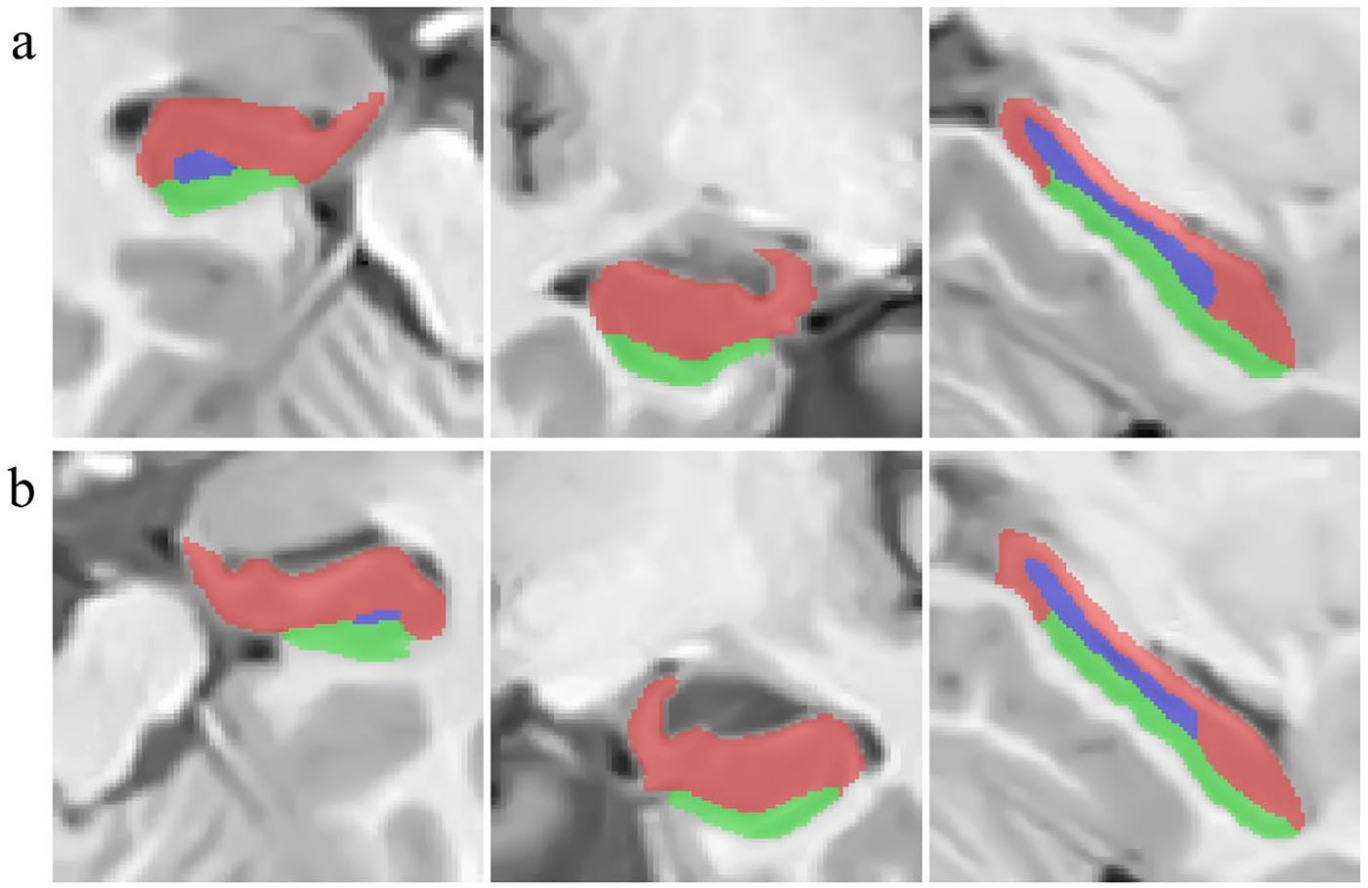
A sample case of images from the left (a) and right (b) hippocampus along with their associated segments (red, Cornu Ammonis areas 1–3 [CA1–CA3] total; blue, Cornu Ammonis area 4 and dentate gyrus [CA4–DG] total; green, subiculum). HIPS: https://www.volbrain.net/services/HIPS).

The hippocampus is a major structure within the limbic system that plays a crucial role in the process of creating new memories and learning. It is divided into the dentate gyrus and cornu ammonis subregions (Y. L. Rao et al. 2022) (Figure 1). The hippocampus is involved in the generation of new neurons in the central nervous system (Zanirati et al. 2023). This structure has been demonstrated to be more vulnerable to damage, possibly due to its fragile blood–brain barrier (Davidson and Stevenson 2024). Moreover, the hippocampus is considered one of the main regions affected by various neurodegenerative disorders, including Alzheimer's disease (AD) (Ghaderi et al. 2025; Weerasinghe‐Mudiyanselage et al. 2022).

AD is a progressive brain disorder that causes cognitive decline and memory loss. Accumulation of extracellular amyloid‐β plaques and hyperphosphorylated tau proteins are the two main pathological hallmarks of AD (Roda et al. 2022). In early‐stage AD, the initial accumulation of tau is observed in the entorhinal cortex, which subsequently spreads to the hippocampus. The hippocampus is then functionally disconnected from other brain regions because of the loss of hippocampal tissue. In the advanced stages of the disease, there is a breakdown in communication between the dentate gyrus and other subfields of the hippocampus, leading to cognitive deficiencies (Y. L. Rao et al. 2022). Imaging analysis of these hippocampal alterations could be a valuable method for enhancing our understanding of the pathological progression of AD.

Advanced magnetic resonance imaging (MRI) techniques such as diffusion tensor imaging (DTI) and diffusion kurtosis imaging (DKI) are becoming more widely used for assessing neurodegenerative disorders (Du et al. 2024). DTI assesses Gaussian diffusion and offers detailed insights into the organization of the brain's white matter at the microstructural level. It assesses the brain microstructure by evaluating the direction and extent of water molecule diffusion within the brain (Assaf and Pasternak 2008). DKI, a modification of DTI, can measure the non‐Gaussian diffusion of water molecules within a voxel, making it well suited for demonstrating the organization of microstructural alterations in both the gray and white matter (Jensen et al. 2005; Jensen and Helpern 2010). It can be used to identify variations in the brain tissue microstructure between patients with AD and cognitively healthy individuals. Additionally, it addresses the limitations of traditional MRI in recognizing microstructural pathological changes before the onset of macroscopic atrophy (Chu et al. 2022).

Several studies have compared DKI metrics between patients with AD and healthy controls (HCs), yielding promising results but not focusing on DKI metrics (G. Rao et al. 2023). Thus, our study aimed to systematically investigate differences in DKI metrics, including mean kurtosis (MK), kurtosis fractional anisotropy (KFA), axial kurtosis (AK), and radial kurtosis (RK), in the hippocampi of patients with AD and HCs. Furthermore, we performed a comprehensive meta‐analysis of MK to evaluate the clinical significance of hippocampal alterations using non‐Gaussian diffusion metrics.

## 2 Methods

### 2.1 Search Strategy

This systematic review and meta‐analysis followed the Preferred Reporting Items for Systematic Reviews and Meta‐Analyses (PRISMA) 2020 guidelines (Page et al. 2021). Five eligible articles did not provide DKI metrics and could not be included, as we did not receive any response to the emails sent (Chen et al. 2017; Cheng et al. 2018; Giraldo et al. 2022; Rallabandi et al. 2023; Struyfs et al. 2015). A systematic search of the PubMed, Scopus, Web of Science, and Embase databases was performed in November 2024 to identify relevant studies. We constructed the search strategy according to the PECO (patients: patients with AD, exposure: DKI, comparison: HCs, outcome: DKI metrics). Keywords related to the patient (AD) and intervention (DKI) components were used, along with appropriate Boolean operators. We also used Medical Subject Headings (MeSH) for our search. The specific search strategies for each database are summarized in Table S1.

### 2.2 Study Selection

**FIGURE 2:**
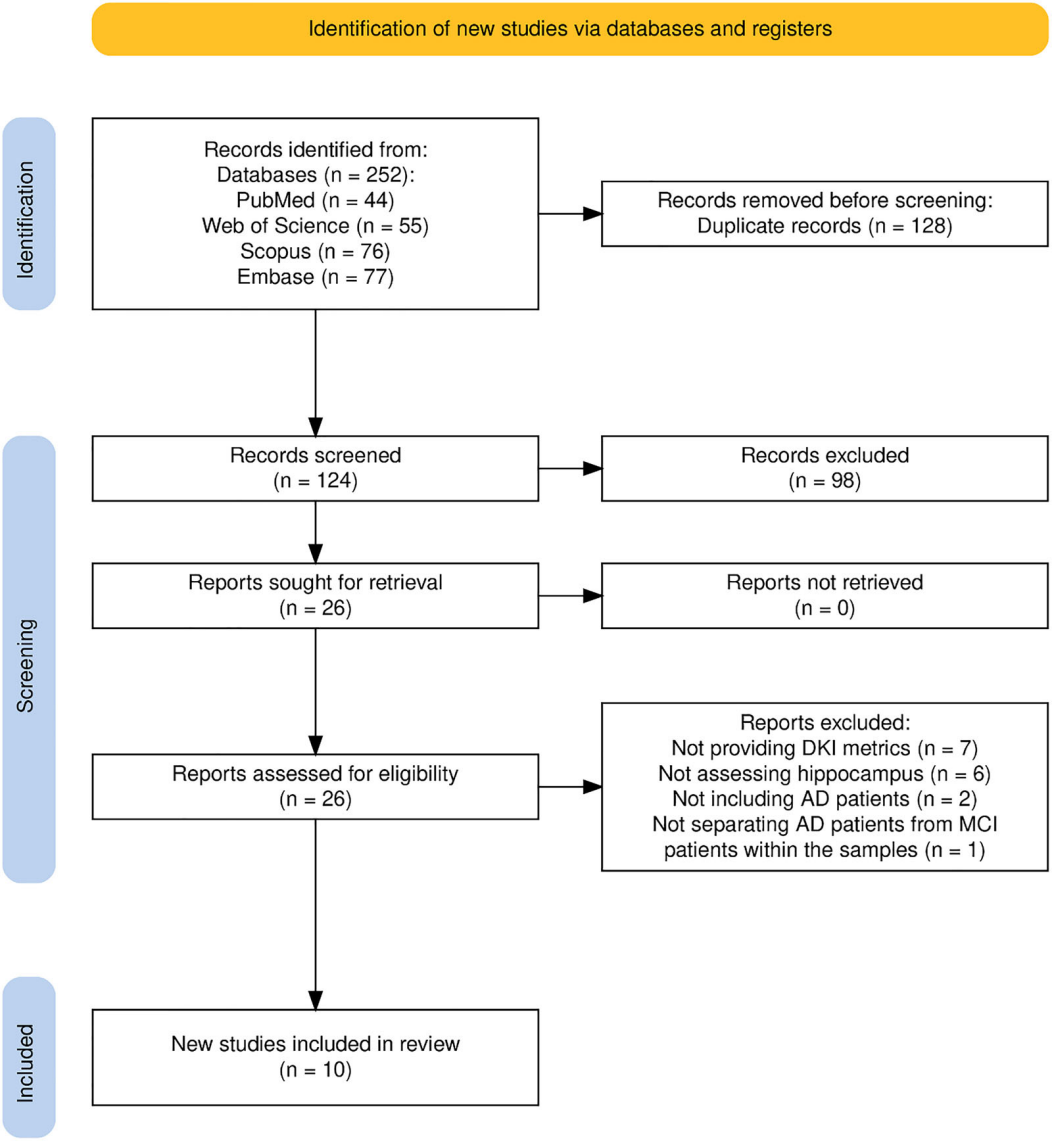
PRISMA flow diagram for study selection process. AD, Alzheimer's disease; DKI, diffusion kurtosis imaging.

We included original works that compared at least one of the hippocampal DKI metrics, including MK, KFA, AK, and RK, between patients with AD, irrespective of disease severity, and normal controls. This study followed a rigorous selection process that adhered to the established standards. The authors ensured high‐quality evidence by including only the studies published in peer‐reviewed journals. Irrelevant articles, case reports/series, conference abstracts, reviews, animal studies, letters/editorials, book chapters, and non‐English‐language studies were excluded. The initially retrieved articles were integrated into the EndNote software. After deduplication, the remaining studies were independently reviewed by two authors (A.M.A. and N.J.) according to their titles and abstracts. The articles selected in this step were then assessed according to their full texts, and the works selected in this second step were reviewed by two experienced authors (S.G. and S.M.). To perform a comprehensive search, we used major databases and manually searched the reference lists of relevant articles. In addition, we sent emails twice to the corresponding authors of the articles that applied DKI to compare AD patients with AD and HCs but did not provide the actual values of DKI metrics for either of these groups. Any discrepancies during the screening process were resolved by a professor of neurology (F.F.), and the final selection of studies was reached through consensus. The study selection process is illustrated in Figure 2.

### 2.3 Data Extraction and Risk of Bias (ROB) Assessment

The data extraction process was meticulously designed to capture relevant information from eligible studies. Two reviewers independently extracted key characteristics, including first author name, publication year, country and continent, subject demographics, MRI characteristics and acquisition parameters, hippocampal DKI metrics, and the main findings. Discrepancies were resolved through discussions.

To assess the quality and potential biases of the included studies, the Newcastle–Ottawa Scale (NOS), which is based on the Ottawa checklist for cross‐sectional studies, was employed (Wells et al. 2000). This widely recognized checklist assesses the quality of studies based on selection, comparability, and outcome criteria, with scores ranging from 0 to 9. A score of 9 represents very good quality, 7–8 good quality, 5–6 satisfactory quality, and 0–4 unsatisfactory quality. “Very good” and “good” ratings indicated a low ROB, whereas others showed a high ROB. “Good” for this review, NOS was adapted slightly following the approach of two previous meta‐analyses (Herzog et al. 2013; Parasuaraman et al. 2023). Three authors (S.M., A.M.A., and N.J.) independently performed the quality assessment, and disagreements were resolved through discussion.

### 2.4 Meta‐Analysis

Following systematic review and data extraction, a meta‐analysis was conducted using STATA software version 17 (StataCorp, College Station, TX, USA). Owing to the lack of sufficient reports for KFA, AK, and RK, we compared only the pooled estimates for MK. The standardized mean difference (SMD) was used to compare MK between patients with AD and HCs. The SMD effect size thresholds (0.2, 0.5, and 0.8) were used to interpret the magnitude of the differences (Cohen 1988). For studies that reported the mean and 95% confidence interval (95% CI) instead of the mean and standard deviation (SD), we calculated the SD using the formula mentioned in the Cochrane Handbook of Systematic Reviews of Interventions on the following website (https://meta‐converter.com/conversions/sd‐ci‐g) (Abbas et al. 2024). A random‐effects model was adopted for all analyses to account for the potential heterogeneity between studies. *I* ^2^ was used to assess heterogeneity with the following cut‐off values: 0%–40%, not important; 30%–60%, moderate heterogeneity; 50%–90%, substantial heterogeneity; and 75%–100%, considerable heterogeneity (Higgins and Thompson 2002).

### 2.5 Subgroup and Sensitivity Analysis

Subgroup analyses were performed to explore the potential sources of heterogeneity and examine the effects of various factors on the DKI metrics of the hippocampus. Subgroup analyses were performed on the basis of male ratio, age, echo time (TE), repetition time (TR), number of directions, and ROB. Sensitivity analysis was also performed using the leave‐one‐out method to evaluate the effect of each study on the pooled estimate and robustness of the overall findings.

### 2.6 Publication Bias Assessment

To assess potential publication bias, funnel plots were first visualized to identify any asymmetry that might suggest selective reporting of studies, followed by quantitative assessment using Egger's regression and Begg's tests (Begg and Mazumdar 1994; Egger et al. 1997). Statistical significance was set at *p* < 0.05. Additionally, trim‐and‐fill analyses were performed for further evaluation.

## 3 Results

### 3.1 Study Characteristics

Among the 252 initially retrieved articles, 10 were included in this systematic review (Chu et al. 2022; Falangola et al. 2013; N. Gong et al. 2017; Niu et al. 2023; Song et al. 2019; M.‐L. Wang et al. 2018; Yuan et al. 2016; Zhang et al. 2023). The total number of samples was 215 (40.47% males) in the AD group and 217 (40.55% males) in the HC group. The mean age ranged from 67.65 to 75.35 years in the AD group and from 65.50 to 73.30 years in the HC group. Disease severity of the included patients ranged from mild to severe (Table 1).

**TABLE 1:** Study characteristics.

References	Country	Continent	Sample size (P)/(male)	Sample size (C)/(male)	Disease stage	Mean age ± SD (y) (P)	Mean age ± SD (y) (C)	MK value ± SD (P)	MK value ± SD (C)	KFA value ± SD (P)	KFA value ± SD (C)	AK value ± SD (P)	AK value ± SD (C)	RK value ± SD (P)	RK value ± SD (C)
Niu et al. (2023)	China	Asia	23/6	37/17	NR	Median (IQR): 72 (63, 81)	Median (IQR): 69 (66.5, 74)	Mean (95% CI) Left Hipp: 0.710 (0.665, 0.754) Right Hipp: 0.760 (0.716, 0.805)	Mean (95% CI) Left Hipp: 0.713 (0.687, 0.739) Right Hipp: 0.790 (0.761, 0.819)	NR	NR	NR	NR	NR	NR
Zhang et al. (2023)	China	Asia	16/5	19/4	NR	75 ± 8.1	73.1 ± 5.9	Left Hipp: 0.6222 ± 0.0408 Right Hipp: 0.6205 ± 0.0871 Total Hipp: 0.6214 ± 0.0546	Left Hipp: 0.7200 ± 0.0773 Right Hipp: 0.7173 ± 0.0670 Total Hipp: 0.7186 ± 0.0627	NR	NR	NR	NR	NR	NR
Chu et al. 2022a()	China	Asia	20/6	20/4	Non‐severe	75.35 ± 8.80	73.30 ± 9.02	Total Hipp: 0.709 ± 0.033	Total Hipp: 0.823 ± 0.042	Hipp: 0.205 ± 0.057	Hipp: 0.346 ± 0.088	Hipp: 0.652 ± 0.102	Hipp: 0.751 ± 0.071	Hipp: 0.713 ± 0.081	Hipp: 0.904 ± 0.125
Raj et al. (2022)	India	Asia	27/12	25/11	NR	68.4 ± 8.4	65.5 ± 7.1	Left Hipp: 0.66 ± 0.07 Right Hipp: 0.64 ± 0.09	Left Hipp: 1.02 ± 0.14 Right Hipp: 1.07 ± 0.17	Left Hipp: 0.19 ± 0.05 Right Hipp: 0.17 ± 0.04	Left Hipp: 0.25 ± 0.07 Right Hipp: 0.24 ± 0.06	Left Hipp: 0.63 ± 0.07 Right Hipp: 0.61 ± 0.09	Left Hipp: 0.75 ± 0.11 Right Hipp: 0.77 ± 0.09	Left Hipp: 0.68 ± 0.08 Right Hipp: 0.66 ± 0.09	Left Hipp: 0.77 ± 0.06 Right Hipp: 0.78 ± 0.06
Song et al. (2019)	China	Asia	20/11	20/11	NR	73.2 ± 4.49	70.45 ± 5.04	Left Hipp: 0.61 ± 0.14 Right Hipp: 0.59 ± 0.10	Left Hipp: 0.79 ± 0.08 Right Hipp: 0.81 ± 0.20	NR	NR	NR	NR	NR	NR
M.‐L. Wang et al. (2018)	China	Asia	Mild AD: 17/7 Moderate‐to‐severe AD: 15/5	16/7	Mild‐to‐severe	Mild AD: 72.6 ± 7.8 Moderate‐to‐severe AD: 74.1 ± 8.6	71.4 ± 7.9	Mild AD: Left Hipp: 0.695 ± 0.045 Right Hipp: 0.710 ± 0.057 Moderate‐to‐severe AD: Left Hipp: 0.667 ± 0.054 Right Hipp: 0.675 ± 0.057	Left Hipp: 0.723 ± 0.033 Right Hipp: 0.723 ± 0.037	NR	NR	NR	NR	NR	NR
N. Gong et al. (2017)	United States	North America	18/7	18/7	Mild‐to‐moderate	73.7 ± 4.2	73.2 ± 5.5	Mean (95% CI): Left Hipp: 0.713 (0.684, 0.743) Right Hipp: 0.729 (0.696, 0.761)	Mean (95% CI): Left Hipp: 0.766 (0.737, 0.795) Right Hipp: 0.764 (0.732, 0.797)	NR	NR	NR	NR	NR	NR
Yuan et al. (2016)	China	Asia	26/12	26/11	Mild	67.65 ± 6.90	65.54 ± 6.04	Left Hipp: 0.70 ± 0.08 Right Hipp: 0.68 ± 0.08	Left Hipp: 0.77 ± 0.03 Right Hipp: 0.79 ± 0.04	NR	NR	Left Hipp: 0.73 ± 0.08 Right Hipp: 0.75 ± 0.08	Left Hipp: 0.71 ± 0.11 Right Hipp: 0.79 ± 0.05	Left Hipp: 0.68 ± 0.08 Right Hipp: 0.71 ± 0.09	Left Hipp: 0.77 ± 0.04 Right Hipp: 0.79 ± 0.04
D. Wang et al. (2015)	China	Asia	20/11	20/11	NR	73.2 ± 4.49	70.45 ± 5.04	Left Hipp: 0.61 ± 0.14 Right Hipp: 0.89 ± 0.10	Left Hipp: 0.79 ± 0.08 Right Hipp: 0.81 ± 0.20	NR	NR	NR	NR	NR	NR
Falangola et al. (2013)	United States	North America	13/5	16/5	Mild‐to‐moderate	75.0 ± 7.4	71.6 ± 7.6	Total Hipp: 0.83 ± 0.02	Total Hipp: 0.87 ± 0.01	NR	NR	Hipp: 0.81 ± 0.02	Hipp: 0.85 ± 0.02	Hipp: 0.85 ± 0.01	Hipp: 0.89 ± 0.01

Abbreviations: AD, Alzheimer's disease; AK, axial kurtosis; C, controls; Hipp, hippocampus; IQR, interquartile range; KFA, kurtosis fractional anisotropy; MK, mean kurtosis; NR, not reported; P, patients; RK, radial kurtosis; SD, standard deviation.

All studies utilized 3.0 Tesla scanners with different coil types ranging from 8 to 64 channels. TEs varied remarkably from 69 ms in the study by N. Gong et al. (2017) to 810 ms in the study by Zhang et al. (2023). TRs also varied widely, from 2000 ms in N. Gong et al. (2017) to 11,500 ms in Raj et al. (2022). The applied sequences included echo‐planar imaging (EPI) (20%), spin‐echo (SE)‐EPI (20%), single‐shot (SS)‐SE‐EPI (20%), twice‐refocused SE‐EPI (10%), and SS twice‐refocused SE‐EPI (10%). Two studies did not report the specific sequences that were applied. To calculate DKI parameters, most studies employed the Diffusion Kurtosis Estimator (DKE) software (70%) (Niu et al. 2023; Zhang et al. 2023; Raj et al. 2022; Chu et al. 2022a; M.‐L. Wang et al. 2018; Yuan et al. 2016; Falangola et al. 2013), whereas two studies applied MRIcron (Song et al. 2019; D. Wang et al. 2015) and another utilized in‐house MATLAB programs (N. Gong et al. 2017). Seven studies used 30 imaging directions, whereas 15, 32, and 64 approaches were applied in one study. Notably, some studies did not report details of certain parameters (Table 2).

**TABLE 2:** Technical characteristics of the included studies.

References	Field strength	Coil	TE (ms)	TR (ms)	Sequence	*b* values	Slice thickness (mm)	Matrix dimensions	Field of view	Voxel size (mm)	Number of directions	Software/Toolbox
Niu et al. (2023)	3	64	72	3800	SE‐EPI	0, 2000, 3000	NR	110 × 110	220 × 220	2 × 2 × 2.2	64	DKE
Zhang et al. (2023)	3	32	810	3900	SS‐SE‐EPI	0, 1000, 2000	3	80 × 80	230 × 90 × 230	NR	15	DKE
Chu et al. ()	3	32	103	2200	NR	0, 1000, 2000	5	192 × 192	220 × 220	0.6 × 0.6 × 5	30	DKE
Raj et al. (2022)	3	32	98	11500	SS twice refocused SE‐EPI	0, 1000, 2000	3	74 × 74	222 × 222	3 × 3	30	DKE and ROI Editor
Song et al. (2019)	3	32	NR	NR	EPI	0, 500, 1000, 1500, 2000, 2500	NR	NR	NR	NR	30	MATLAB and MRIcron
M.‐L. Wang et al. (2018)	3	32	109	5400	SE‐EPI	0, 500, 1000, 1500, 2000, 2500	3	128 × 128	230	NR	30	DKE
N. Gong et al. (2017)	3	8	69	2000	NR	0, 1000, 2000	NR	128 × 128	NR	2 × 2 × 3	32	MATLAB
Yuan et al. (2016)	3	32	103	10500	SS‐SE‐EPI	0, 1000, 2000	NR	128 × 128	230 × 230	1.8 × 1.8 × 1.8	30	DKE
D. Wang et al. (2015)	3	32	109	1100	EPI	0, 500, 1000, 1500, 2000, 2500	4	128 × 128	300	2.3 × 2.3 × 4	30	MRIcron
Falangola et al. (2013)	3	NR	109	2300	Twice‐refocused SE‐EPI	0, 500, 1000, 1500, 2000, 2500	2	128 × 128	256 × 256	2 × 2 × 2	30	DKE

Abbreviations: DKE, Diffusion Kurtosis Estimator; EPI, echo‐planar imaging; mm, millimeter; ms, millisecond; NR, not reported; SE, spin‐echo; SS, single‐shot.

All included studies reported MK in both AD and HC groups. The ranges of the mean MK for the total hippocampus, left hippocampus, and right hippocampus were 0.62–0.83, 0.61–0.71, and 0.59–0.89, respectively. Only two studies evaluated KFA, one for the total hippocampus and the other for both the left and right hippocampi. Furthermore, four studies assessed AK and RK, two assessed the total hippocampus, and two assessed the left and right hippocampus (Table 1).

### 3.2 Quality Assessment

The results of the overall quality assessment are presented in Table 3. Among the eight studies included, four were categorized as having high ROB, with a total score of 6. All four studies had selection bias in the non‐respondent domain. Conversely, the remaining six studies, with scores ranging from 7 to 8, were deemed to have a low ROB. All studies received scores corresponding to the representativeness of the sample, sample size, ascertainment of exposure (risk factor), assessment of the outcome, and statistical test domains. Regarding the control for important or additional factor domains, six studies scored 1, and the others scored 2.

**TABLE 3:** Details of quality assessment according to the Newcastle–Ottawa Scale (NOS) adapted to the cross‐sectional studies.

References	Selection				Comparability	Outcome		Total score	Quality
	Representativeness of the sample	Sample size	Non‐respondents	Ascertainment of the exposure (risk factor)	Control for important or additional factors	Assessment of the outcome	Statistical test		
Niu et al. (2023)	1	1	0	1	1	1	1	6	High ROB
Zhang et al. (2023)	1	1	1	1	1	1	1	7	Low ROB
Chu et al. ()	1	1	0	1	1	1	1	6	High ROB
Raj et al. (2022)	1	1	1	1	1	1	1	7	Low ROB
Song et al. (2019)	1	1	0	1	1	1	1	6	High ROB
M.‐L. Wang et al. (2018)	1	1	1	1	2	1	1	8	Low ROB
N. Gong et al. (2017)	1	1	1 L | 0 R	1	2	1	1	8 L | 7 R	Low ROB
Yuan et al. (2016)	1	1	1	1	2	1	1	8	Low ROB
D. Wang et al. (2015)	1	1	1	1	2	1	1	8	Low ROB
Falangola et al. (2013)	1	1	0	1	1	1	1	6	High ROB

Abbreviations: L, left; R, right; ROB, risk of bias.

### 3.3 Meta‐Analysis

**FIGURE 3:**
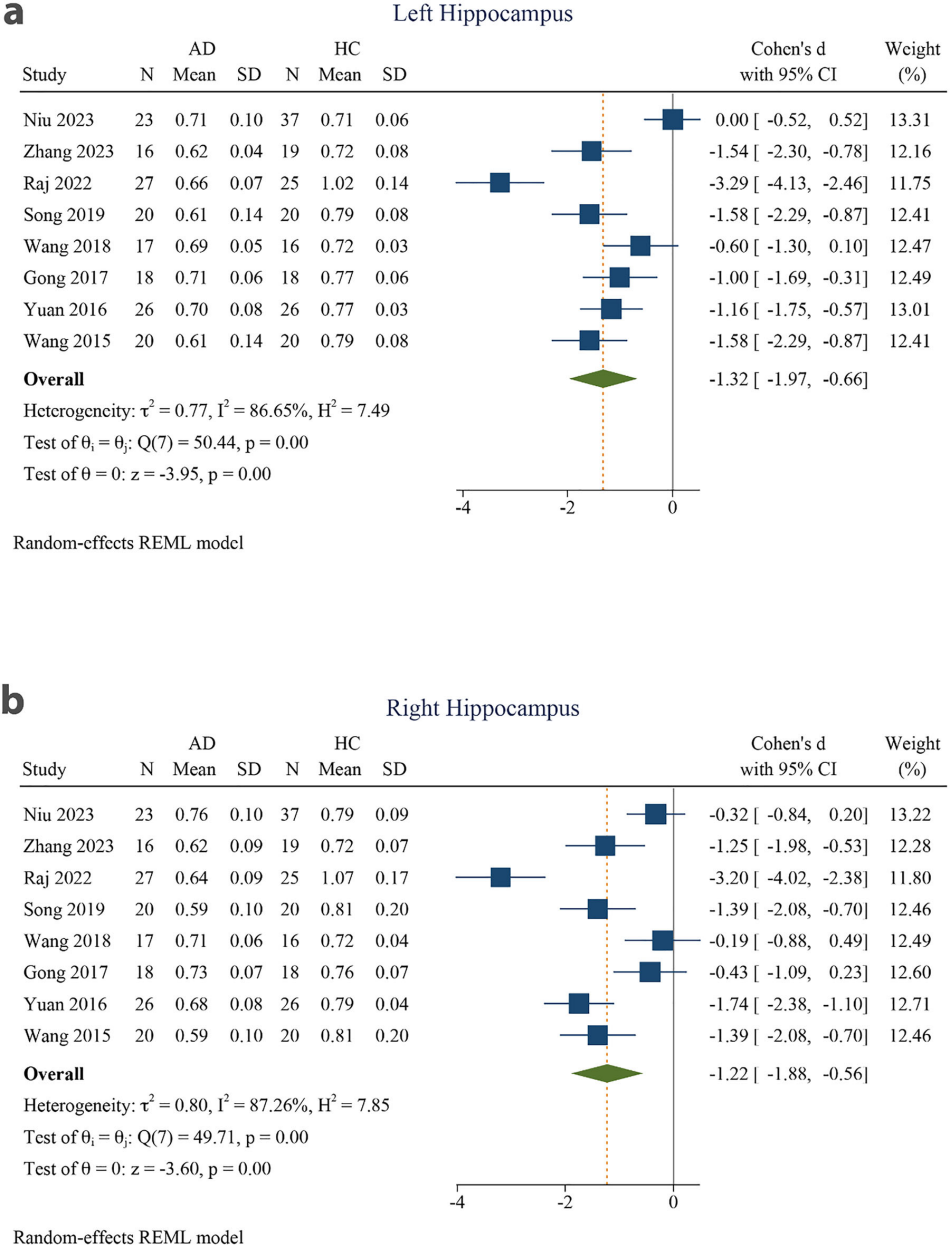
Meta‐analysis of mean kurtosis in the (a) left hippocampus and (b) right hippocampus of Alzheimer's disease patients compared to healthy controls (*τ* ^2^: the variance observed between different studies; *H* index: the ratio of the variance between studies to the variance within studies, serving as an indicator of heterogeneity; *Q* index: Cochrane *Q*‐test, which assesses heterogeneity through a statistical method; *I* ^2^: an indicator representing the proportion of total variation among studies attributed to heterogeneity instead of random chance. The test of *θ_i_* = *θ* evaluates the homogeneity of effect sizes across various studies (where *θ_i_* and *θ_j_* denote the effect sizes from the two distinct studies). The *θ* = 0 test was employed to analyze the overall effect observed across all studies (with *θ* representing the overall effect size). 95% CI, 95% confidence interval; AD, Alzheimer's disease; HC, healthy control.

Eight articles were included in this meta‐analysis (Niu et al. 2023; Zhang et al. 2023; Raj et al. 2022; Song et al. 2019; M.‐L. Wang et al. 2018; N. Gong et al. 2017; Yuan et al. 2016; D. Wang et al. 2015). On the basis of these reports, we performed separate analyses of the left and right hippocampus. All analyses, including subgroup analyses, revealed decreased MK values in the AD group compared to the HC group for both the left and right hippocampi (Table 4). There was a significant SMD in the MK of AD patients and the HC group in the left hippocampus (−1.32 [−1.97 to −0.66], *p* < 0.001) (Figure 3a). The SMD was also significant for the right hippocampus (−1.22 [−1.88 to −0.56], *p* < 0.001). The pooled effect size was large for both the left and right hippocampus. There was substantial to considerable heterogeneity among reports for both the left (*I* ^2^ = 86.65%) and right hippocampi (*I* ^2^ = 87.26%) (Figure 3b).

**TABLE 4:** Summary of meta‐analysis and subgroup analysis results.

Variable	*K*	Left hippocampus				Right hippocampus			
		SMD (95% CI)	*p* value	*P*_heterogeneity_	*I* ^2^%	SMD (95% CI)	*p* value	*P*_heterogeneity_	*I* ^2^%
Overall	8	−1.32 (−1.97 to −0.66)	*p* < 0.001	<0.001	86.65	−1.22 (−1.88 to −0.56)	<0.001	<0.001	87.26
Male ratio									
>42%	4	−1.87 (−2.77 to −0.97)	0.05	<0.001	84.79	−1.91 (−2.72 to −1.10)	<0.001	<0.001	80.97
≤42%	4	−0.78 (−1.40 to −0.10)		0.01	74.25	−0.52 (−0.94 to −0.09)		0.14	43.06
Age									
>72	5	−1.25 (−1.64 to −0.86)	0.83	0.20	34.02	−0.93 (−1.43 to −0.42)	0.36	0.03	62.93
≤72	3	−1.46 (−3.34 to 0.41)		<0.001	96.21	−1.73 (−3.35 to −0.10)		<0.001	94.66
TE									
Long (>100)	4	−1.21 (−1.63 to −0.78)	0.84	0.19	35.11	−1.15 (−1.81 to −0.49)	0.88	0.01	73.02
Short (≤100)	3	−1.41 (−3.31 to 0.49)		<0.001	95.83	−1.30 (−3.12 to 0.53)		<0.001	95.67
TR									
Long (>5000)	3	−1.67 (−3.25 to −0.08)	0.46	<0.001	93.50	−1.70 (−3.39 to −0.01)	0.33	<0.001	94.10
Short (≤5000)	4	−1.28 (−2.03 to −0.53)		<0.001	80.57	−0.82 (−1.36 to −0.27)		0.03	65.17
Number of directions									
>30	2	−0.47 (−1.45 to 0.51)	0.07	0.02	80.45	−0.36 (−0.77 to 0.05)	0.01	0.80	0.00
≤30	6	−1.60 (−2.30 to −0.91)		<0.001	82.67	−1.51 (−2.27 to −0.75)		<0.001	85.64
Risk of bias									
High	2	−0.77 (−2.32 to 0.78)	0.40	<0.001	91.91	−0.83 (−1.88 to 0.22)	0.45	0.02	82.98
Low	6	−1.32 (−1.97 to −0.66)		<0.001	84.08	−1.35 (−2.20 to −0.51)		<0.001	88.52

Abbreviations: SMD, standardized mean difference; TE, echo time; TR, repetition time.

### 3.4 Male Ratio Subgroup

**FIGURE 4:**
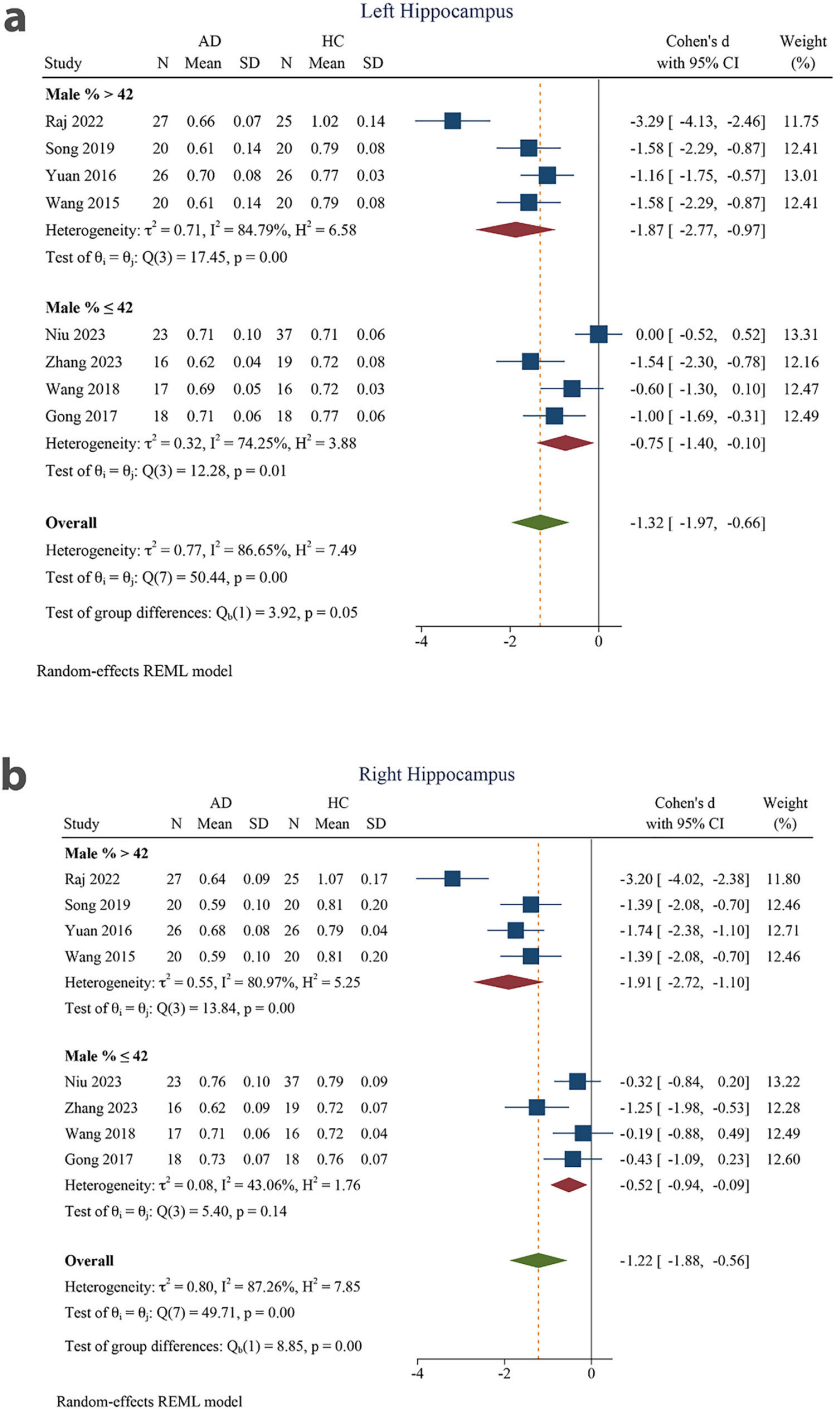
Subgroup analysis based on male ratio for (a) left hippocampus and (b) right hippocampus. 95% CI, 95% confidence interval; AD, Alzheimer's disease; HC, healthy control.

Subgrouping studies based on the male ratio revealed a large pooled effect size for both the left (SMD = −1.87 [−2.77 to −0.97]) and right (SMD = −1.91 [−2.72 to −1.10]) hippocampi in the male ratio >42% subgroups. Within the male ratio ≤42% subgroups, a moderate‐to‐large pooled effect size was found for both the left (SMD = −0.75 [−1.40 to −0.10]) and right (SMD = −0.52 [−0.94 to −0.09]) hippocampi. The SMD was significant in both subgroups of the male ratio for both the left and right hippocampus. The intergroup difference was marginally significant in the left hippocampus (*p* = 0.05) and significant in the right hippocampus (*p* < 0.001) (Figure 4).

### 3.5 Age Subgroup

**FIGURE 5:**
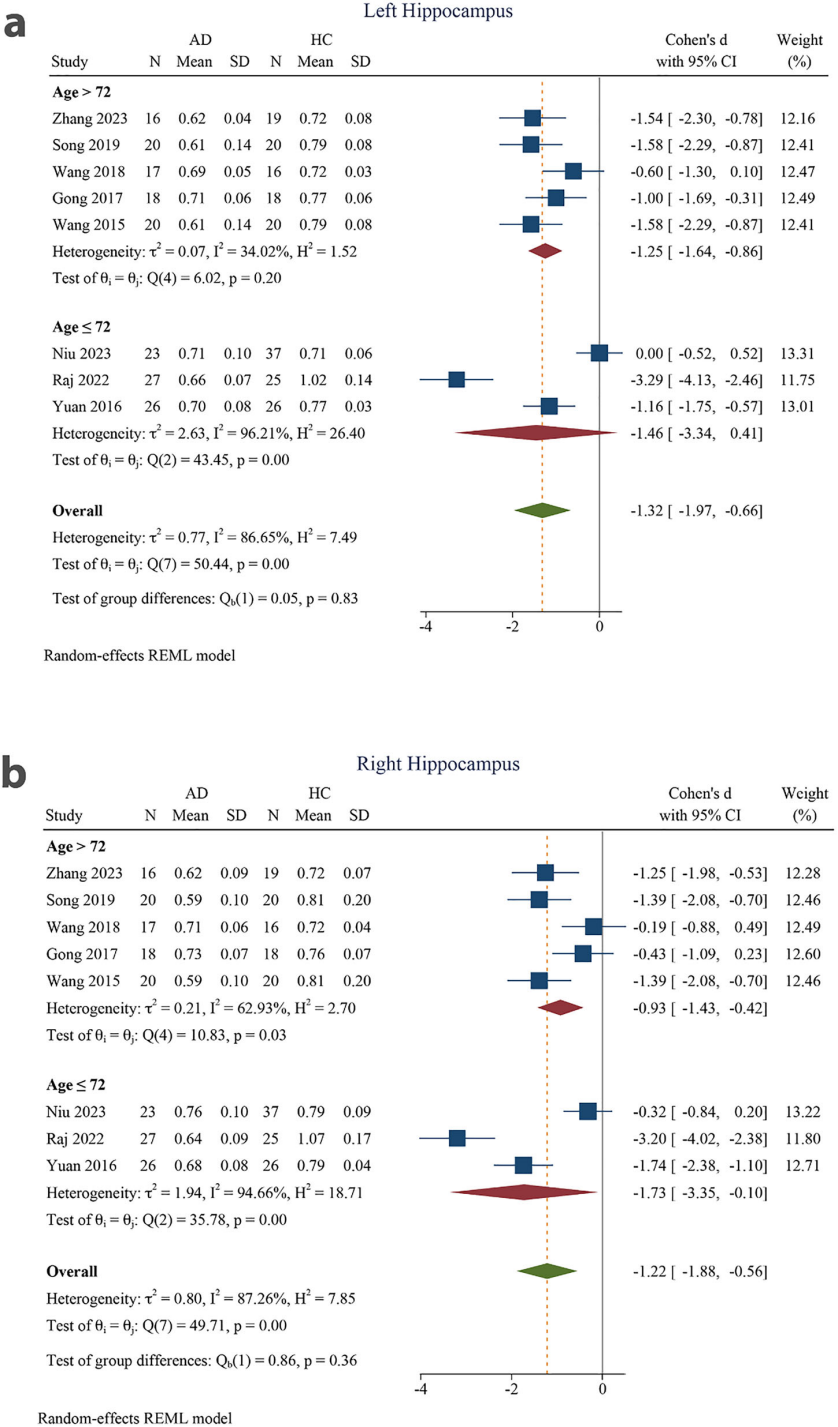
Subgroup analysis based on mean age for (a) left hippocampus and (b) right hippocampus. 95% CI, 95% confidence interval; AD, Alzheimer's disease; HC, healthy control.

The pooled effect size was large for both the left (SMD = −1.25 [−1.64 to −0.86]) and right (SMD = −0.93 [−1.43 to −0.42]) hippocampi in the age >72 years subgroup. Similarly, the pooled effect size was large for both the left (SMD = −1.46 [−3.34 to 0.41]) and right (SMD = −1.73 [−3.35 to −0.10]) hippocampi in the age ≤72 years subgroups. The SMD was significant in the age >72 years subgroup for both the left and right hippocampi and in the age ≤72 years subgroup for the right hippocampus, but not in the age ≤72 years subgroup for the left hippocampus. There were no significant inter‐group differences (*p* = 0.83 in the left and *p* = 0.36 for the right hippocampus) (Figure 5).

### 3.6 Echo Time Subgroup

**FIGURE 6:**
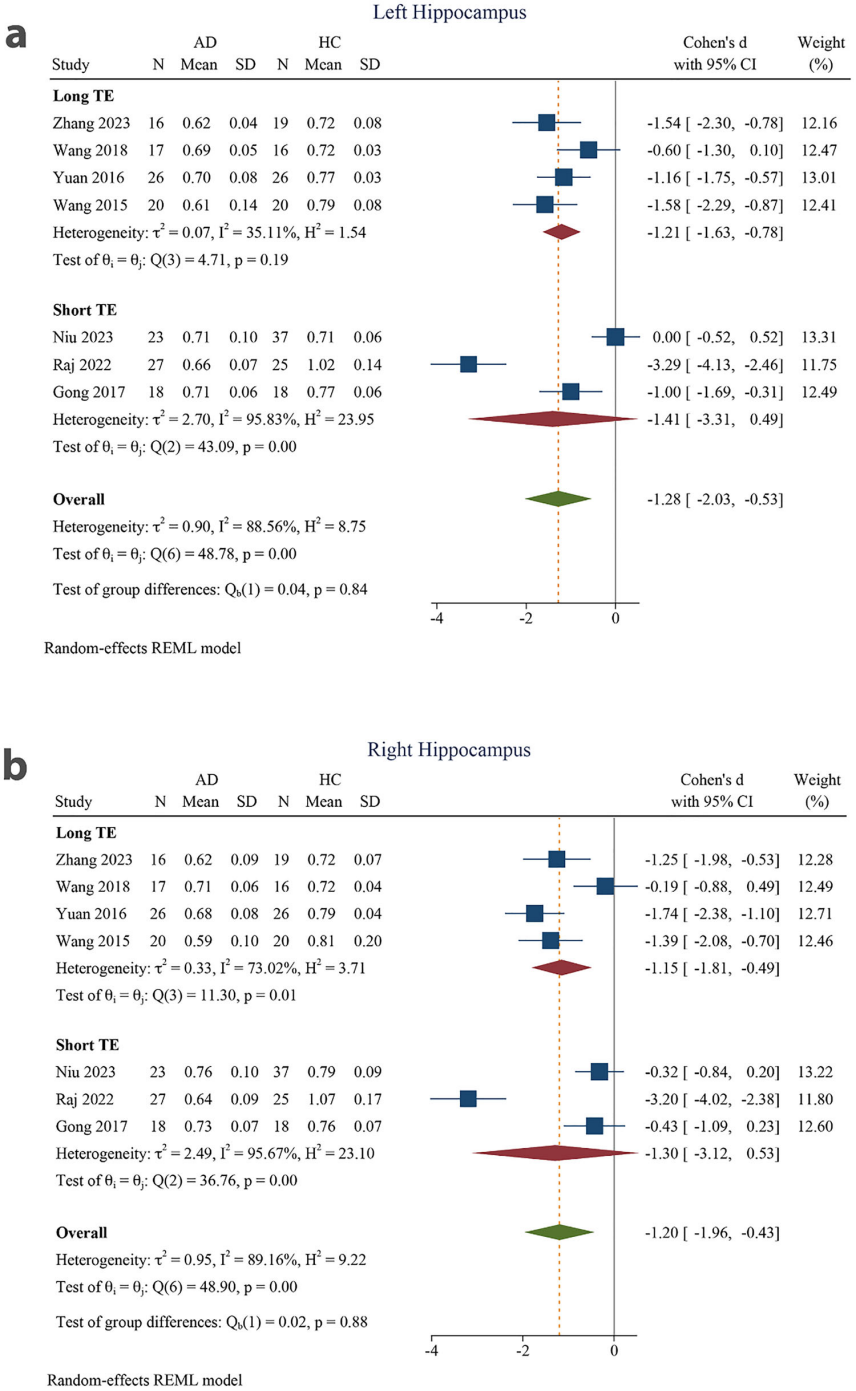
Subgroup analysis based on echo time (TE) for (a) left hippocampus and (b) right hippocampus (long TE >100 and short TE ≤100). 95% CI, 95% confidence interval; AD, Alzheimer's disease; HC, healthy control.

The pooled effect size was large for both the left (SMD = −1.21 [−1.63 to −0.78]) and right (SMD = −1.15 [−1.81 to −0.49]) hippocampi within the long TE subgroups. Moreover, the pooled effect size was large for both the left (SMD = −1.41 [−3.31 to 0.49]) and right (SMD = −1.30 [−3.12 to 0.53]) hippocampi within the short TE subgroups. The SMD was significant in the long TE subgroups for both the left and right hippocampi, but not in the short TE subgroups. Intergroup differences were not significant for the left (*p* = 0.84) or right hippocampus (*p* = 0.88) (Figure 6).

### 3.7 Repetition Time Subgroup

**FIGURE 7:**
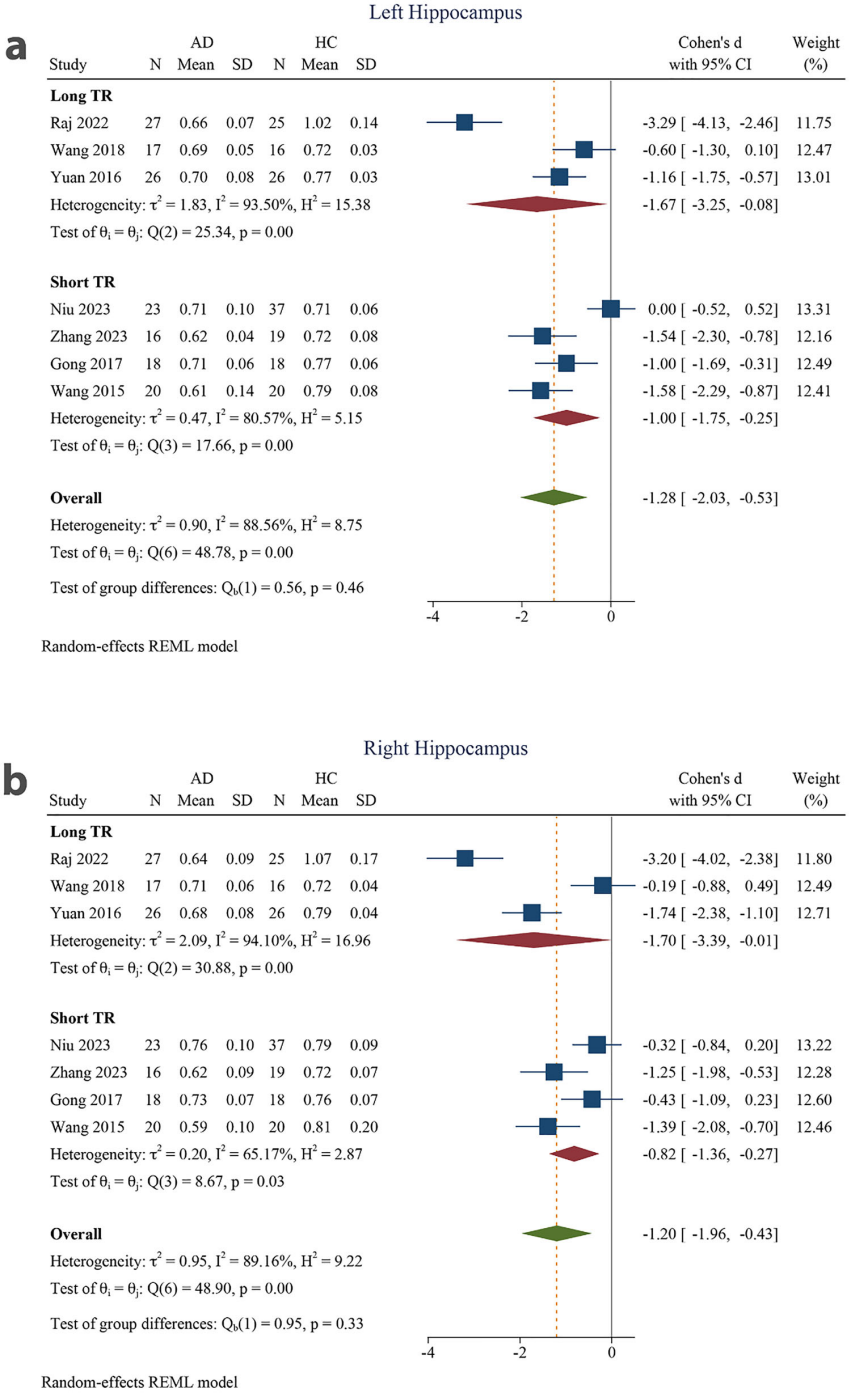
Subgroup analysis based on repetition time (TR) for (a) left hippocampus and (b) right hippocampus (long TR >5000 and short TR ≤5000). 95% CI, 95% confidence interval; AD, Alzheimer's disease; HC, healthy control.

The subgroup analyses based on TR revealed a significant SMD and a large pooled effect size for both the left and right hippocampi within both the long and short TR subgroups (SMD = −1.67 [−3.25 to −0.08] for the left hippocampus in the long TR subgroup, SMD = −1.70 [−3.39 to −0.01] for the right hippocampus in the long TR subgroup, SMD = −1.00 [−1.75 to −0.25] for the left hippocampus in the short TR subgroup, and SMD = −0.82 [−1.36 to −0.27] for the right hippocampus in the short TR subgroup). Intergroup differences were not significant for the left (*p* = 0.46) or right hippocampus (*p* = 0.33) (Figure 7).

### 3.8 Number of Directions Subgroup

**FIGURE 8:**
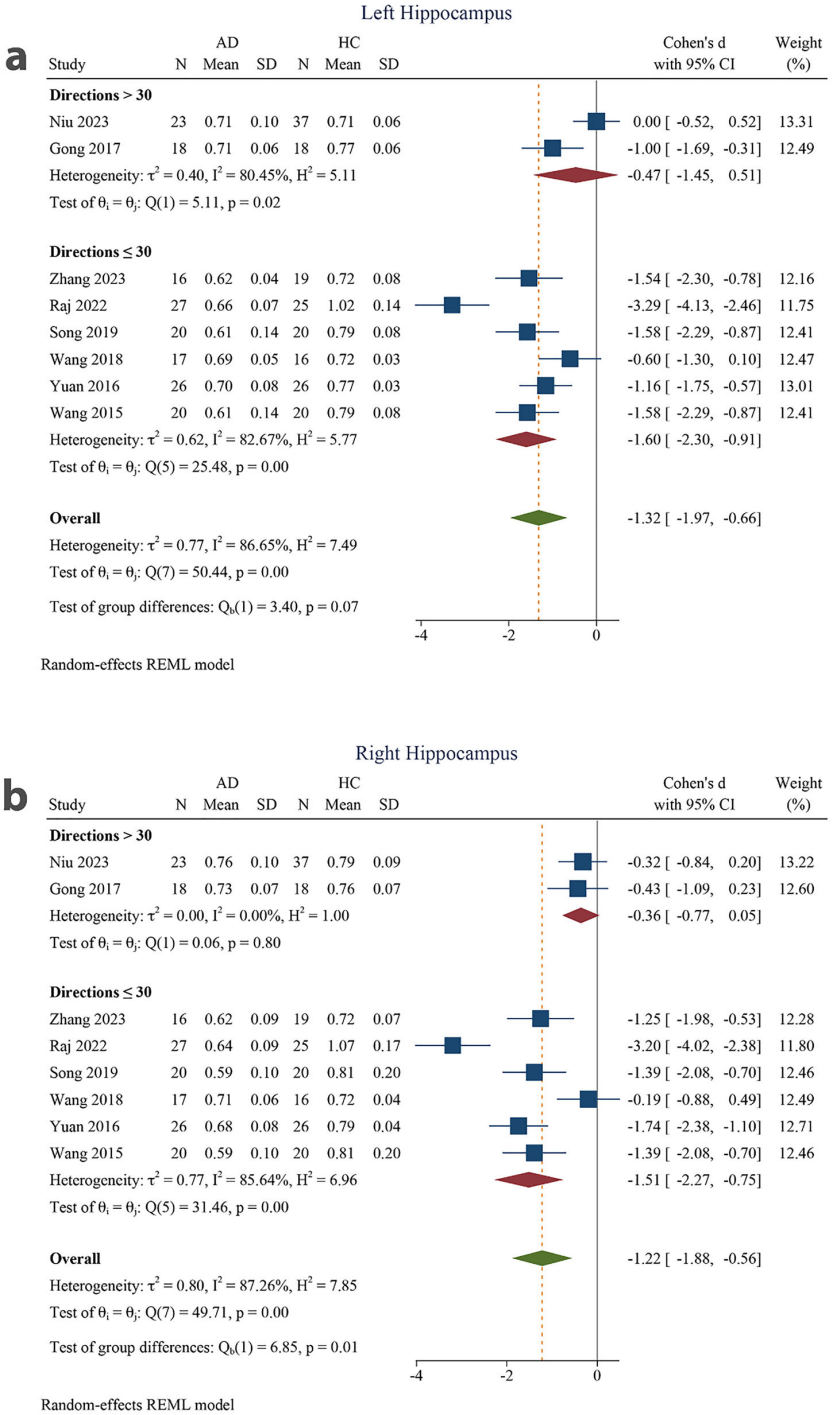
Subgroup analysis based on number of directions for (a) left hippocampus and (b) right hippocampus. 95% CI, 95% confidence interval; AD, Alzheimer's disease; HC, healthy control.

The subgroup analysis showed a large pooled effect size for both the left (SMD = −1.60 [−2.30 to −0.91]) and right (SMD = −1.51 [−2.27 to −0.75]) hippocampi in the number of imaging directions ≤30 subgroups and a small‐to‐moderate pooled effect size for both the left (SMD = −0.47 [−1.45 to 0.51]) and right (SMD = −0.36 [−0.77 to 0.05]) hippocampi in the number of imaging directions >30 subgroups. The SMD was not significant in the number of imaging directions >30 subgroups for both the left and right hippocampi, whereas it was significant for ≤30 imaging directions. The intergroup difference was not significant in the left hippocampus (*p* = 0.07), but it was significant in the right hippocampus (*p* = 0.01) (Figure 8).

### 3.9 ROB Subgroup

**FIGURE 9:**
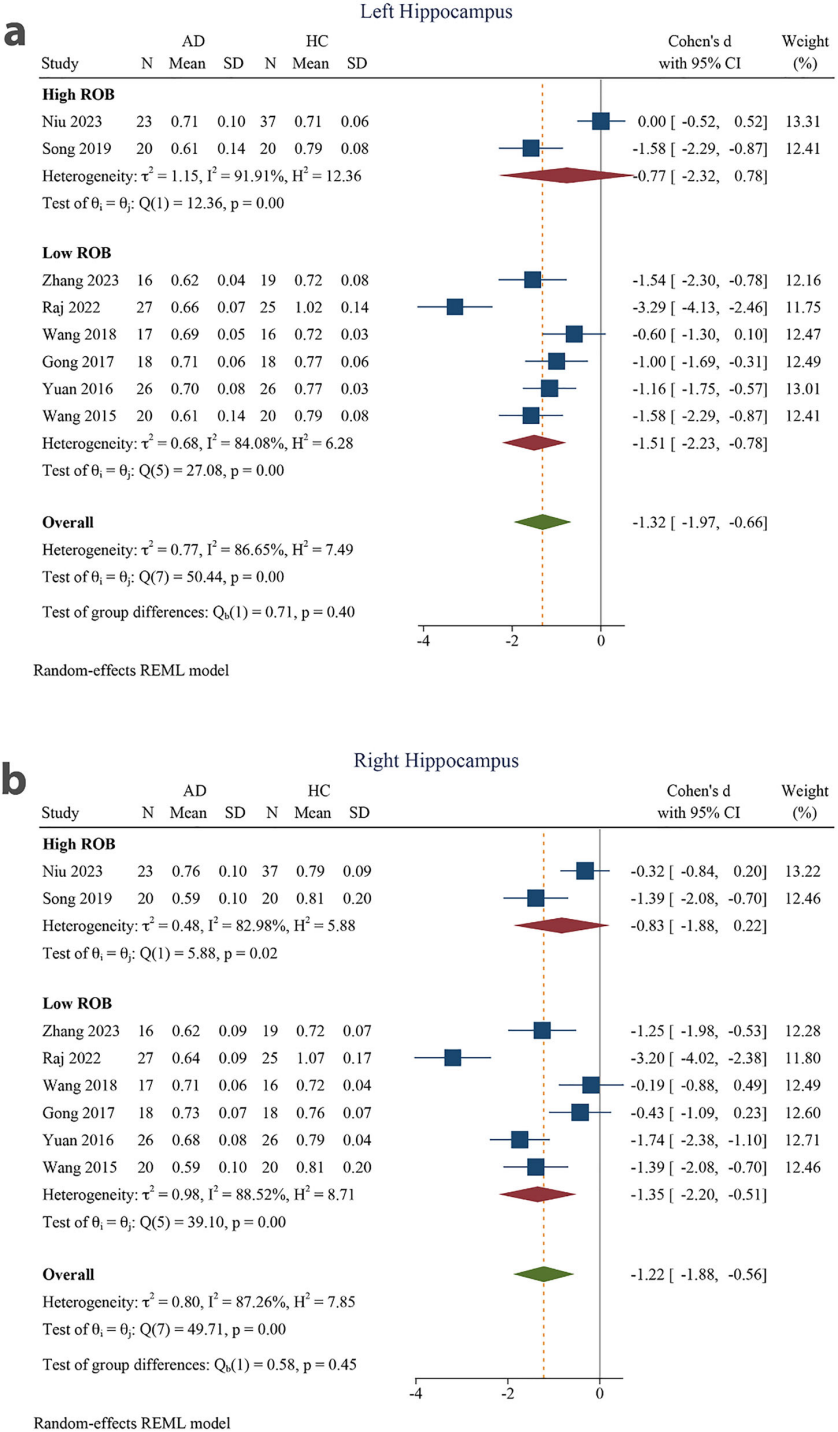
Subgroup analysis based on risk of bias (ROB) for (a) left hippocampus and (b) right hippocampus. 95% CI, 95% confidence interval; AD, Alzheimer's disease; HC, healthy control.

Subgroup analysis revealed a non‐significant SMD in the subgroups of studies that had a high ROB for both the left and right hippocampi. In contrast, the SMD was significant, and the pooled effect size was large for studies with a low ROB. Among the studies with a low ROB, the SMD was −1.51 [−2.23 to −0.78] for the left hippocampus and −1.35 [−2.20 to −0.51] for the right hippocampus. These subgroup analyses did not yield a significant intergroup difference (*p* = 0.40 for the left and *p* = 0.45 for the right hippocampus (Figure 9).

### 3.10 Sensitivity Analysis

**FIGURE 10:**
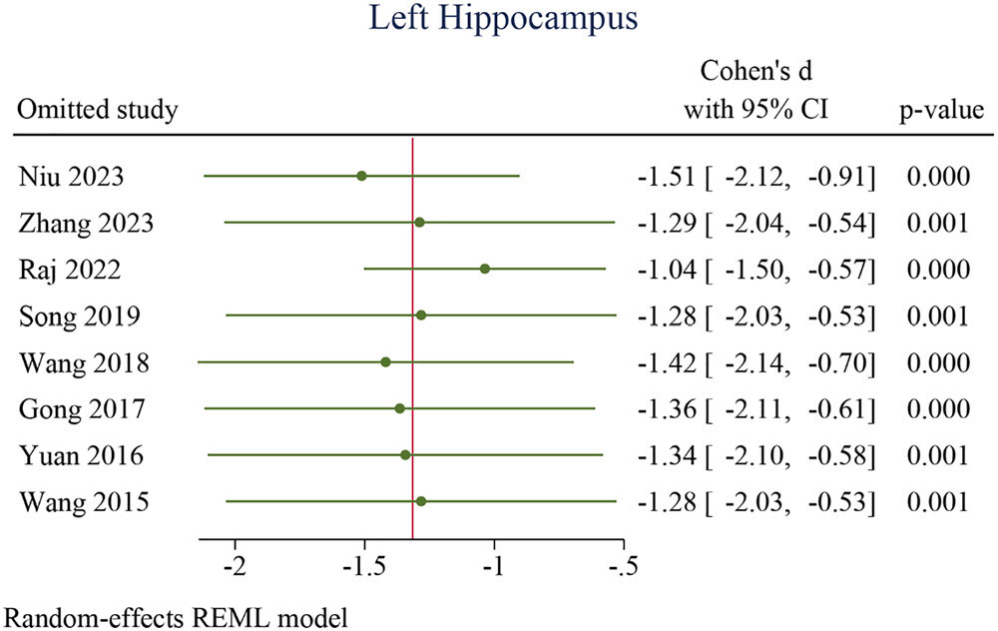
Sensitivity analysis for left hippocampus. 95% CI, 95% confidence interval.

**FIGURE 11:**
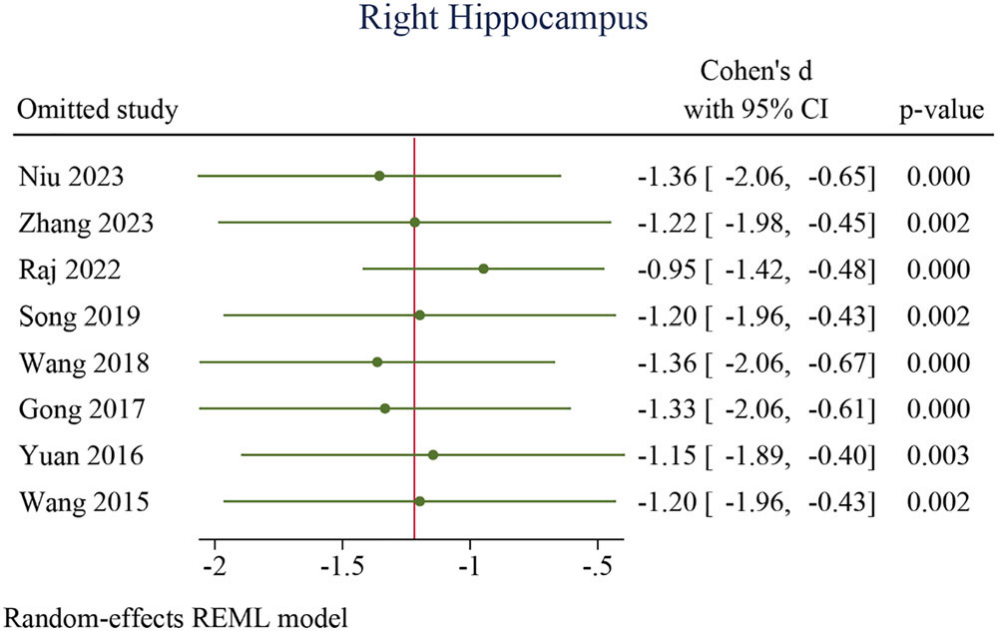
Sensitivity analysis for right hippocampus. 95% CI, 95% confidence interval.

For the left hippocampus (Figure 10), omitting individual studies one at a time yielded Cohen's *d* values ranging from −1.04 (95% CI: −1.50 to −0.57) to −1.51 (95% CI: −2.12 to −0.91), with all *p* values ≤0.001. Similarly, for the right hippocampus (Figure 11), effect sizes remained stable across iterations, ranging from −0.95 (95% CI: −1.42 to −0.48) to −1.36 (95% CI: −2.06 to −0.65), with *p* values ≤0.003. In both regions, confidence intervals for all omitted studies excluded zero, and effect estimates retained consistent direction and magnitude. These results confirm that no study disproportionately influenced the pooled effects, underscoring the robustness of microstructural hippocampal alterations observed in AD.

### 3.11 Publication Bias

Egger's regression test for the left side revealed significant small‐study effects (*β* = −15.25, *z* = −3.85, *p* < 0.001), a finding supported by Begg's rank correlation test (Kendall's score = −17.00, *z* = −2.26, *p* = 0.044). However, the trim‐and‐fill analysis identified no missing studies (observed = 8, imputed = 0), with minimal impact on the pooled effect size (Figure S1). For the right side, Egger's test also indicated significant small‐study effects (*β* = −15.77, *z* = −2.57, *p* = 0.010), though Begg's test did not reach statistical significance (Kendall's score = −11.00, *z* = −1.51, *p* = 0.209). Similarly, the trim‐and‐fill analysis detected no imputed studies (observed = 8, imputed = 0), and the pooled effect size remained consistent (Figure S2). Taken together, these findings suggest potential asymmetry in smaller studies. The mixed results may reflect limited statistical power or heterogeneity in study characteristics, but no substantial evidence of actionable publication bias was identified.

## 4 Discussion

Our systematic review and meta‐analysis aimed to elucidate microstructural alterations in the hippocampus of patients with AD. Our findings, supported by the robustness of the sensitivity analysis and the assessment of ROB and publication bias, revealed decreased MK values in the bilateral hippocampi of patients with AD compared with HCs. This is consistent with previous research and suggests that MK is a sensitive biomarker of microstructural changes related to water diffusion in the hippocampus in AD (N.‐J. Gong et al. 2013; Struyfs et al. 2015). MK reflects the complexity and heterogeneity of the microstructural compositions within the brain tissue. Reduced MK values indicate decreased structural complexity or heterogeneity, likely due to neuronal degeneration, atrophy, and apoptosis, which leads to increased extracellular diffusion space (Andica et al. 2020; Arab et al. 2018; Fan et al. 2022; D. Wang et al. 2015).

Moreover, the observed decrease in MK values in patients with AD aligns with the known pathological features of AD, including neuronal loss, synaptic degeneration, and accumulation of amyloid‐β plaques and tau tangles (DeTure and Dickson 2019; Falcon et al. 2018). The hippocampus is a critical brain region for memory and learning, and its dysfunction is a hallmark of AD (Y. L. Rao et al. 2022). Hippocampal atrophy is commonly observed in AD and mild cognitive impairment (Feng et al. 2021; Horn et al. 1996; Josephs et al. 2017; Pennanen et al. 2004; Rusinek et al. 2004; Schuff et al. 2009), but minor microstructural changes detectable by diffusion‐based biomarkers may occur even before macroscopic volume loss becomes apparent (Hong et al. 2010; Shahid et al. 2022; van Uden et al. 2015), particularly in DKI‐based biomarkers (Matsumoto et al. 2025).

Our results are consistent with those of previous studies that reported microstructural abnormalities in the hippocampus of patients with AD using other multimodal and multiparametric‐based neuroimaging analyses (Hong et al. 2010; Lee et al. 2017; Moallemian et al. 2023; Zhao et al. 2022). However, the use of DKI provides a more nuanced understanding of these alterations by capturing non‐Gaussian diffusion properties that are not detectable by conventional DTI in AD and other neurodegenerative disorders (Allen et al. 2019; Chen et al. 2017; Ghaderi et al. 2024; Mohammadi et al. 2025; Raj et al. 2022; Rallabandi et al. 2023; Yuan et al. 2016). This advantage of DKI over DTI highlights its potential for early detection of AD‐related changes before the onset of macroscopic atrophy (Yuan et al. 2016; Zhang et al. 2023). Overall, this suggests a reduction in the complexity and non‐Gaussian diffusion of water molecules within the hippocampus, potentially reflecting alterations in cellularity, myelination, and neuronal integrity (Karat et al. 2024).

The MK reductions were influenced by clinical and technical factors such as the male ratio, age, TE, TR, and the number of diffusion directions. This suggests potential sex‐ and age‐related influences on the hippocampal microstructure in AD (Reas et al. 2021; Schweitzer et al. 2023) and indicates that specific acquisition parameters can influence the sensitivity of DKI in detecting AD‐related hippocampal alterations (Cheung et al. 2009; Comrie et al. 2024; Grinberg et al. 2017). After additional computations, our subgroup analysis indicated that TR was a major source of heterogeneity in the left hippocampal analysis in all the studies. Other factors such as sex, direction, age, TE, and ROB assessment can contribute to heterogeneity, albeit to a lesser extent. In the analysis of the right hippocampus, the sources of heterogeneity were age, TR, TE, sex, ROB assessment, and direction.

A male‐to‐female ratio greater than 42% revealed that the reduction in MK within the hippocampus might be more pronounced in AD cohorts with a higher proportion of male participants. Recent studies have reported that males have a higher overall mid‐life dementia risk but a lower risk of late‐life AD than females (Brady et al. 2024). However, we found inconsistent literature on this matter (Beam et al. 2018; Lopez‐Lee et al. 2024; Podcasy and Epperson 2016), and the reasons for the more prominent MK reduction observed in male‐dominant subgroups remain unclear and warrant further investigation. It is crucial to consider other factors that may have contributed to this observation, such as the potential sex‐related differences in disease progression, brain structure, and genetics.

Although the SMD for the bilateral hippocampus was large in our two subgroup analyses based on the mean age of patients with AD, the subgroup analysis based on age revealed a non‐significant difference in the pooled effect size for MK between the AD cohorts with a mean age greater than 72 years and those with a mean age less than or equal to 72 years. This finding suggests that age may not be a significant factor influencing the magnitude of MK reduction in the hippocampus of patients with AD. Although brain atrophy is associated with both normal aging and AD (D. Wang et al. 2013), solely using structural MRI to accurately differentiate normal atrophy from AD atrophy, especially in the early stages of the disease, can be challenging (Fjell et al. 2009). A longitudinal study demonstrated that the hippocampus in normal aging reduces by approximately 1%–2% annually, compared to 3%–4% in AD (Fjell et al. 2009). This highlights the need for longitudinal studies with longer follow‐up periods to enhance the statistical power of sMRI for determining abnormal atrophy. Differentiating the rate and pattern of atrophy between normal aging and AD requires more comprehensive investigation, and age alone may not be sufficient to explain the variance in MK values observed.

Subgroup analyses based on parameter acquisitions, including TE, TR, and the number of diffusion directions, did not reveal significant differences in the SMD for MK between subgroups. This suggests that variations in these technical parameters may not substantially influence the magnitude of the MK reduction observed in the hippocampi of patients with AD. However, it is important to acknowledge that a study exploring the application of various MRI modalities in AD research noted that inconsistent findings in DTI studies could be attributed to factors such as scanning parameters and different post‐processing methods (Stebbins and Murphy 2009). Despite the non‐significant findings in the subgroup analyses, future research should carefully consider and standardize these technical parameters to ensure the reliability and comparability of the DKI findings across different studies (Steven et al. 2014). Further investigation of the potential impact of these technical factors, particularly in conjunction with other variables, may provide a more comprehensive understanding of their influence on MK measurements in AD.

Our subgroup analysis based on the number of diffusion‐encoding directions yielded a noteworthy result. Studies employing 30 or fewer directions reported a significant and large pooled effect size, whereas the subgroup with more than 30 directions did not show a significant difference, particularly for the right hippocampus, where the intergroup difference was significant. This finding may seem counterintuitive, as a higher number of diffusion directions is generally expected to improve the accuracy and stability of kurtosis tensor estimation. However, this result is likely attributable to a lack of statistical power in the high‐direction subgroup, which consisted of only two studies. The small sample size in this subgroup may have led to a Type II error, failing to detect a true effect. Although our analysis does not conclusively demonstrate a benefit for a higher number of directions, it highlights a critical source of heterogeneity in the literature and emphasizes the urgent need for standardized DKI acquisition protocols to ensure that findings are comparable and reproducible across studies.

A critical finding from our subgroup analysis was the moderating effect of study quality, as assessed by the ROB. Studies classified as having a low ROB demonstrated a large and statistically significant reduction in MK in both the left (SMD = −1.51) and right (SMD = −1.35) hippocampi. In contrast, the analysis of studies with a high ROB yielded a non‐significant effect. This disparity underscores the profound impact of methodological rigor on meta‐analytic outcomes in neuroimaging. The sources of bias in the lower quality studies, such as potential selection bias or inadequate control for confounding variables, may have introduced noise or systematic errors that obscured the true effect size. Therefore, the robust findings from the low‐ROB studies likely provide a more accurate estimate of the true microstructural hippocampal degradation in AD, strengthening our overall conclusion while simultaneously cautioning that the reliability of DKI as a biomarker is contingent upon high‐quality study design and execution.

## 5 Limitations and Future Direction

This study had several limitations that must be acknowledged. The significant heterogeneity observed in the meta‐analysis (*I* ^2^ = 86.65% for the left hippocampus and *I* ^2^ = 87.26% for the right hippocampus) likely stems from variations in study methodologies, including imaging protocols (TE, TR, and number of diffusion directions), sample characteristics (age, sex, and disease severity), and data processing techniques. This heterogeneity may have influenced the pooled effect sizes and necessitated cautious interpretation of the results. Despite the high heterogeneity, the significantly lower MK values in AD patients compared to HCs were also found in the age >72 and long TE subgroups for the left hippocampus (subgroups with *I* ^2^ < 40). Furthermore, the cross‐sectional nature of the included studies precludes inferences regarding causal relationships between hippocampal microstructural changes and AD progression.

The potential impact of differences in DKI acquisition protocols and post‐processing pipelines on pooled results is significant. Variations in *b* values, the number and orientation of diffusion gradients, TE, TR, and the choice of kurtosis fitting algorithms can substantially influence MK measurements. Such methodological variability may contribute to observed heterogeneity and complicate the interpretation of pooled effect sizes. Specifically, studies employing higher maximum *b* values or advanced noise‐correction approaches may produce systematically different MK estimates compared to those using conventional acquisition schemes, potentially biasing combined results toward certain parameter ranges. This highlights the need for future multi‐center DKI studies to adopt standardized acquisition and processing protocols to enhance cross‐study comparability and reproducibility.

The potential impact of differences in DKI acquisition protocols and post‐processing pipelines on the pooled results. Variations in *b* values, the number and orientation of diffusion gradients, TE, TR, and the choice of kurtosis fitting algorithms can all substantially influence MK measurements. Such methodological variability may contribute to the observed heterogeneity and complicate the interpretation of pooled effect sizes. In particular, studies using higher maximum *b* values or advanced noise‐correction approaches may produce systematically different MK estimates compared to those employing conventional acquisition schemes, potentially biasing the combined results toward certain parameter ranges. This underscores the need for future multi‐center DKI studies to adopt standardized acquisition and processing protocols to improve cross‐study comparability and reproducibility.

Although asymmetry was detected in both hippocampi (stronger for the left), the lack of imputed studies in trim‐and‐fill implies minimal impact of publication bias on effect estimates. Caution is warranted in interpreting small‐study effects, particularly for the left hippocampus, where bidirectional tests (Egger and Begg) aligned. These results support the robustness of the meta‐analytic findings for microstructural hippocampal alterations in AD.

Although our review focused on the hippocampus as a whole, it is known that subfields such as CA1‐4, dentate gyrus, subiculum, and hippocampal strata (stratum radiatum, lacunosum, and moleculare [SRLM]) may be differentially affected in early and late stages of AD, with distinct patterns of microstructural degeneration. MK, as a composite diffusion metric, may reflect a complex interplay of axonal, dendritic, and glial alterations that vary spatially within the hippocampus. However, most included studies lacked subfield‐level resolution, limiting our ability to identify more localized patterns of change. Future DKI studies leveraging high‐resolution acquisitions and hippocampal segmentation could help disentangle these spatially specific microstructural alterations, potentially offering greater sensitivity to early disease stages and disease progression.

The limited data availability for DKI metrics beyond MK restricted our analysis, highlighting the need for future investigations exploring KFA, AK, and RK to provide a more comprehensive understanding of hippocampal microstructural alterations. Future research should prioritize longitudinal studies with larger, more diverse cohorts to validate these findings, investigate the temporal dynamics of MK changes in disease progression, and explore the relationship between hippocampal MK values and cognitive performance. Combining DKI with other imaging modalities such as structural MRI, amyloid PET, and tau PET would elucidate the interplay between microstructural changes, macroscopic atrophy, and pathological protein accumulation. Investigating hippocampal MK as a biomarker for early diagnosis and monitoring of treatment response is also warranted. If future research confirms the robustness and clinical utility of hippocampal MK, DKI can be integrated into the diagnostic workup and management of AD. Early detection of microstructural changes could potentially enable earlier interventions, potentially slowing disease progression and improving patient outcomes. Although this meta‐analysis suggests that hippocampal MK is a promising marker of AD, further research is crucial to establish its definitive role in the diagnosis, prognosis, and treatment of AD.

## 6 Conclusions

This systematic review and meta‐analysis synthesized the current evidence on hippocampal microstructural alterations in AD using DKI. Our meta‐analysis of MK, the most frequently reported DKI metric, revealed significant reductions in patients with AD compared to HCs, indicating compromised microstructural integrity within the hippocampus. Notably, a higher proportion of male participants was associated with a more prominent MK reduction, suggesting a potential sex‐related difference in the magnitude of the microstructural changes. Although age, TE, TR, and the number of diffusion directions did not significantly influence SMD, future research should prioritize methodological rigor and standardized technical parameters to enhance the reliability and comparability of DKI findings.

## Author Contributions

**Amir Mahmoud Ahmadzadeh**: investigation, methodology, validation, writing – original draft, project administration, formal analysis, visualization, data curation. **Sadegh Ghaderi**: conceptualization, methodology, validation, visualization, writing – review and editing, writing – original draft, investigation, software, formal analysis, project administration, supervision, data curation. **Sana Mohammadi**: investigation, writing – original draft, methodology, writing – review and editing, validation, software, formal analysis, data curation. **Nahid Jashirenezhad**: writing – original draft, investigation, validation, visualization, data curation. **Farzad Fatehi**: writing – review and editing, supervision.

## Ethics Statement

The authors have nothing to report.

## Conflicts of Interest

The authors declare no conflicts of interest.

## Peer Review

The peer review history for this article is available at https://publons.com/publon/10.1002/brb3.70919

## Supporting information




**Supplementary Material**: brb370919‐sup‐0001‐SuppMat.docx

## Data Availability

This article contains all the data produced or analyzed during this investigation. Further inquiries should be forwarded to the corresponding author.
