# Supplementary material for: Microstructural Hippocampal Alterations in Alzheimer's Disease: A Systematic Review and Meta‐Analysis of Diffusion Kurtosis Imaging
Source: Brain Behav. 2025 Sep 21;15(9):e70919. doi: 10.1002/brb3.70919 (PMC12451067; doi:10.1002/brb3.70919)
Supplement: Supplementary file 1 — Supplementary Material: brb370919‐sup‐0001‐SuppMat.docx [file BRB3-15-e70919-s001.docx]

| **Supplementary Table 1.** The search strategies used for database searches | | |
| --- | --- | --- |
|  | **Database** | **N** |
| PubMed | ("Alzheimer*"[All Fields] OR "Alzheimer’s"[All Fields] OR "Alzheimer's"[All Fields] OR "Alzheimer Syndrome"[All Fields] OR "Alzheimer-Type Dementia (ATD)"[All Fields] OR "Dementia, Alzheimer-Type (ATD)"[All Fields] OR "Alzheimer's Diseases"[All Fields] OR "Alzheimer Dementia"[All Fields] OR "Dementia, Alzheimer"[All Fields] OR "Alzheimer's Disease"[All Fields] OR "Dementia, Senile"[All Fields] OR "Senile Dementia"[All Fields] OR "Dementia, Alzheimer Type"[All Fields] OR "Alzheimer Type Dementia"[All Fields] OR "Senile Dementia, Alzheimer Type"[All Fields] OR "Alzheimer Type Senile Dementia"[All Fields] OR "Primary Senile Degenerative Dementia"[All Fields] OR "Alzheimer Sclerosis"[All Fields] OR "Sclerosis, Alzheimer"[All Fields] OR "Dementia, Primary Senile Degenerative"[All Fields] OR "Dementia, Presenile"[All Fields] OR "Presenile Dementia"[All Fields] OR "Acute Confusional Senile Dementia"[All Fields] OR "Senile Dementia, Acute Confusional"[All Fields] OR "Alzheimer Disease, Early Onset"[All Fields] OR "Early Onset Alzheimer Disease"[All Fields] OR "Presenile Alzheimer Dementia"[All Fields] OR "Alzheimer Disease, Late Onset"[All Fields] OR "Late Onset Alzheimer Disease"[All Fields] OR "Alzheimer's Disease, Focal Onset"[All Fields] OR "Focal Onset Alzheimer's Disease"[All Fields] OR "Familial Alzheimer Disease (FAD)"[All Fields] OR "Alzheimer Disease, Familial (FAD)"[All Fields] OR "Familial Alzheimer Diseases (FAD)"[All Fields]) AND ("Diffusion kurtosis imag*"[All Fields] OR DKI[All Fields]) | 44 |
| Scopus | TITLE-ABS-KEY ( ( "alzheimer&apos;s " OR " alzheimer AND syndrome " OR " alzheimer-type AND dementia ( atd ) " OR " dementia, AND alzheimer-type ( atd ) " OR " alzheimer&apos;s AND diseases " OR " alzheimer AND dementia " OR " dementia, AND alzheimer " OR " alzheimer&apos;s AND disease " OR " dementia, AND senile " OR " senile AND dementia " OR " dementia, AND alzheimer AND type " OR " alzheimer AND type AND dementia " OR " senile AND dementia, AND alzheimer AND type " OR " alzheimer AND type AND senile AND dementia " OR " primary AND senile AND degenerative AND dementia " OR " alzheimer AND sclerosis " OR " sclerosis, AND alzheimer " OR " dementia, AND primary AND senile AND degenerative " OR " dementia, AND presenile " OR " presenile AND dementia " OR " acute AND confusional AND senile AND dementia " OR " senile AND dementia, AND acute AND confusional " OR " alzheimer AND disease, AND early AND onset " OR " early AND onset AND alzheimer AND disease " OR " presenile AND alzheimer AND dementia " OR " alzheimer AND disease, AND late AND onset " OR " late AND onset AND alzheimer AND disease " OR " alzheimer&apos;s AND disease, AND focal AND onset " OR " focal AND onset AND alzheimer&apos;s AND disease " OR " familial AND alzheimer AND disease ( fad ) " OR " alzheimer AND disease, AND familial ( fad ) " OR " familial AND alzheimer AND diseases ( fad ) " ) AND ( diffusion AND kurtosis AND imaging OR dki ) ) | 76 |
| Web of Science (WOS) | ALL=(("Alzheimer's" OR "Alzheimer Syndrome" OR "Alzheimer-Type Dementia (ATD)" OR "Dementia, Alzheimer-Type (ATD)" OR "Alzheimer's Diseases" OR "Alzheimer Dementia" OR "Dementia, Alzheimer" OR "Alzheimer's Disease" OR "Dementia, Senile" OR "Senile Dementia" OR "Dementia, Alzheimer Type" OR "Alzheimer Type Dementia" OR "Senile Dementia, Alzheimer Type" OR "Alzheimer Type Senile Dementia" OR "Primary Senile Degenerative Dementia" OR "Alzheimer Sclerosis" OR "Sclerosis, Alzheimer" OR "Dementia, Primary Senile Degenerative" OR "Dementia, Presenile" OR "Presenile Dementia" OR "Acute Confusional Senile Dementia" OR "Senile Dementia, Acute Confusional" OR "Alzheimer Disease, Early Onset" OR "Early Onset Alzheimer Disease" OR "Presenile Alzheimer Dementia" OR "Alzheimer Disease, Late Onset" OR "Late Onset Alzheimer Disease" OR "Alzheimer's Disease, Focal Onset" OR "Focal Onset Alzheimer's Disease" OR "Familial Alzheimer Disease (FAD)" OR "Alzheimer Disease, Familial (FAD)" OR "Familial Alzheimer Diseases (FAD)") AND ("Diffusion kurtosis imaging" OR DKI)) | 55 |
| Embase | (alzheimer* OR alzheimers:ab OR "Alzheimer Syndrome":ab OR "Alzheimer-Type Dementia (ATD)":ab OR "dementia, Alzheimer-Type (ATD)":ab OR "Alzheimers Diseases":ab OR "Alzheimer Dementia":ab OR "Dementia, Alzheimer":ab OR "Alzheimers Disease":ab OR "Dementia, Senile":ab OR "Senile Dementia":ab OR "Dementia, Alzheimer Type":ab OR "Alzheimer Type Dementia":ab OR "Senile Dementia, Alzheimer Type":ab OR "Alzheimer Type Senile Dementia":ab OR "Primary Senile Degenerative Dementia":ab OR "Alzheimer Sclerosis":ab OR "Sclerosis, Alzheimer":ab OR "Dementia, Primary Senile Degenerative":ab OR "Dementia, Presenile":ab OR "Presenile Dementia":ab OR "Acute Confusional Senile Dementia":ab OR "Senile Dementia, Acute Confusional":ab OR "Alzheimer Disease, Early Onset":ab OR "Early Onset Alzheimer Disease":ab OR "Presenile Alzheimer Dementia":ab OR "Alzheimer Disease, Late Onset":ab OR "Late Onset Alzheimer Disease":ab OR "Alzheimers Disease, Focal Onset":ab OR "Focal Onset Alzheimers Disease":ab OR "Familial Alzheimer Disease (FAD)":ab OR "Alzheimer Disease, Familial (FAD)":ab OR "Familial Alzheimer Diseases (FAD)":ab) AND ("Diffusion kurtosis imag*":ab OR DKI:ab) | 77 |

**
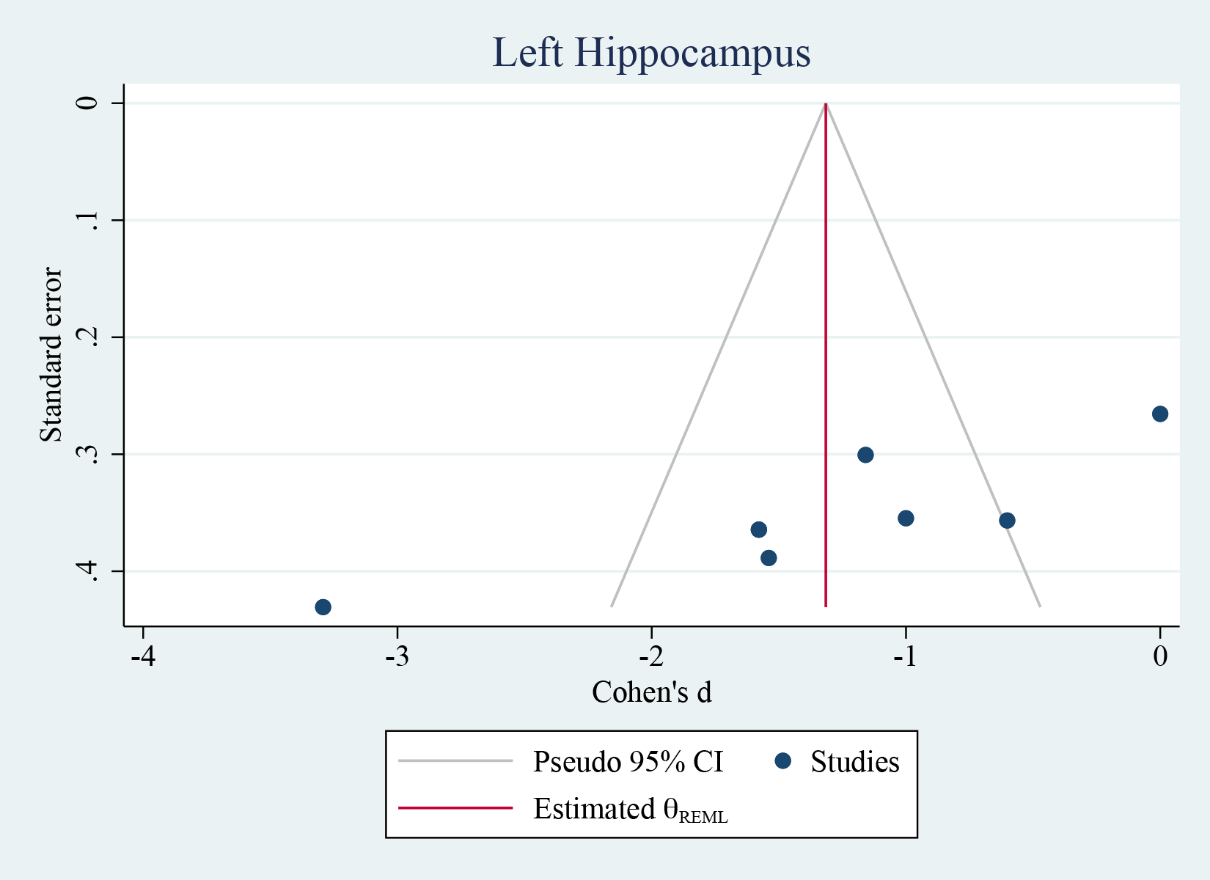
**

**Supplementary Figure 1.** Left hippocampus funnel plot with Trim-and-Fill analysis


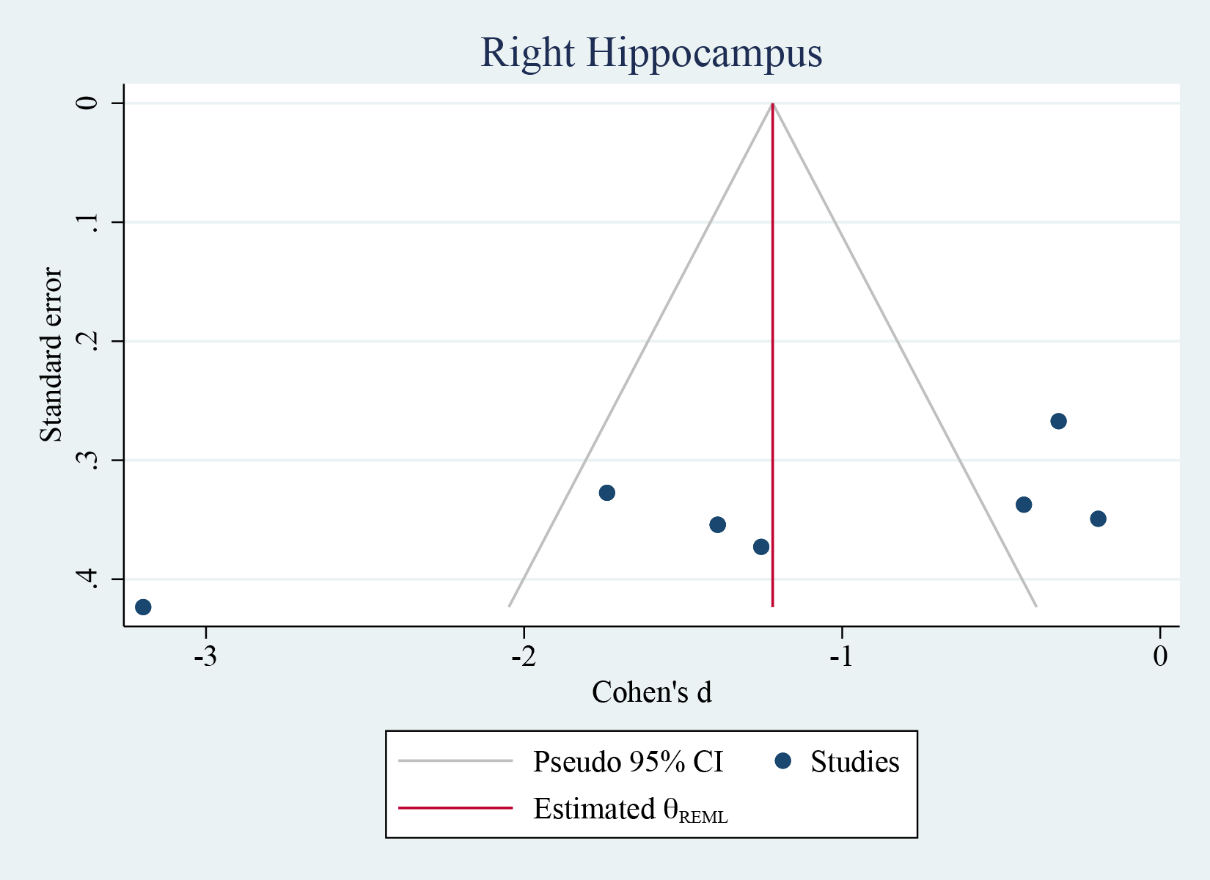


**Supplementary Figure 2.** Right hippocampus funnel plot with Trim-and-Fill analysis
